# Equity-diversity-inclusion (EDI)-related strategies used by dental schools during the admission/selection process: a narrative review

**DOI:** 10.1038/s41405-024-00233-4

**Published:** 2024-07-03

**Authors:** Diego Machado Ardenghi, Renata Grazziotin-Soares, Silvana Papagerakis, Petros Papagerakis

**Affiliations:** 1 Faculty of Dentistry, University of British Columbia, Vancouver, BC Canada; 2 Faculty of Dentistry, Laval University, Quebec City, QC Canada

**Keywords:** Dentistry, Dental education

## Abstract

**Introduction:**

Decades of evidence have demonstrated a lack of workforce diversity and sustaining disparities in academic dentistry and professional practice. Underrepresented minority students may face challenges and implicit bias during the dental schools‘ admission/selection process. This review collected papers from different countries to summarize the Equity-Diversity-Inclusion (EDI)-related strategies that dental schools worldwide have used in their admissions process to increase diversity.

**Methods:**

A comprehensive search using MEDLINE (via PubMed), ERIC, Cochrane Reviews, Cochrane Trials, American Psychological Association Psyc Info (EBSCO) and Scopus was done between January and March-2023. All types of articles-designs were included, except comments and editorials, and all articles selected were in English. Two independent investigators screened the articles. Extracted data were general characteristics, study objectives, and EDI-related strategies.

**Results:**

Sixteen publications were used to construct this manuscript. The year with the greatest number of publications was 2022. Type of studies were case studies/critical reviews (50%), cross-sectional (including survey and secondary data analysis) (*n* = 5, 31.25%), qualitative methods of analysis (*n* = 2, 12.5%), and retrospective/secondary data collection (n = 1, 6.25%). The strategies described in the articles were related to (1) considering the intersectionality of diversity, (2) using noncognitive indicators during the school admissions process to construct a holistic selection process, (3) diversifying, professionalizing, and providing training to admissions persons who had leadership roles with the support from the dental school and the university, and (4) allocating financial investments and analyzing current policies and procedures regarding EDI.

**Conclusions:**

This review aggregated interesting findings, such as: some schools are considering the intersectionality of diversity as a way to include underrepresented minorities and to diversify the students-body. The recent growth in publications on EDI during dental admission/selection process might indicate a positive movement in this field.

## Introduction

It is well established that higher education institutions in the health care field (including dental schools) may be exclusionary and racialized organizations with longstanding structures that prioritize the dominant gender, racial, and ethnic groups [1]. This issue negatively impacts the careers of underrepresented population in dentistry [2]. Evidence have demonstrated a lack of workforce diversity and sustaining disparities in academic dentistry and professional practice—which ultimately affects patient care and oral health outcomes in our society [3–5].

In several countries the organizations responsible for the accreditation process of dental schools have encouraged actions related to equity, diversity, and inclusion (EDI) [6, 7]. One of the reasons for this is that diverse ideas, talent, perspectives, and experiences can build an innovative, prosperous, and inclusive academic community [8]. Therefore, schools have given priority to increase the representation of diverse students in their dentistry programs. However, some applicants still face barriers and discrimination during the admissions selection process [9]. Currently, dental schools are responsible for embracing and increasing EDI to advance the dental profession further, and to serve the communities under their care [7, 10, 11].

The literature only describes the practices that have been implemented independently by single institutions. There is no article that comprehensively aggregate the strategies used by dental schools worldwide. Considering the restricted amount of available evidence and the distinctive study designs, this current narrative review was created. We aimed to answer the following question: What are the EDI-related strategies used by dental schools during their admission/selection process to increase diversity?

## Methods

### Articles search & concepts

This review searched the past and current strategies (i.e., a plan, idea, action, or method of doing something) that have been used by dental schools during their admission/selection process. The investigated strategies were the ones related to increasing diversity and/or underrepresented minorities students in the dental program (BDS, DDS, or DMD). The researchers of this paper conducted an initial search in PubMed and Google Scholar to assess the status of the available literature. Based on the outcome of interest we identified approximately 8 articles that could be included on this review. Up to date, no manuscript has comprehensively described the strategies or best practices made worldwide to diversify dental education. The identified articles independently reported (i.e., for each dental school separately) the actions and ideas used during the recruitment of students and throughout the application/admission/selection process. For this review, diversity was defined in terms of gender, racial and ethnic identities. Underrepresented minorities were defined as groups that have disproportionately lower numbers of dentists compared to the general population. These terms were used as synonyms and interchangeable. This review included both qualitative and quantitative studies.

### Search strategy & data extraction

Two independent researchers executed a search of the literature in the following databases: PubMed (no date restriction), ERIC (no date restriction), Cochrane Reviews (Issue 2, February 2023), Cochrane Trials (Issue 2, February 2023), American Psychological Association Psyc Info (EBSCO) (2000 to date), and Scopus (no date limit). In case of any disagreements, discussions took place to reach an agreement, and a third researcher was included, if necessary. We used keywords and controlled vocabulary where appropriate to describe gender, racial and ethnic minorities, diversity, and recruitment initiatives concepts for each of the databases searched. Keywords and vocabulary were collected from two major documents: Mabeza et al. [12]., and Thorne et al. [13].. This review did not include indigenous/aboriginals (or other related descriptors). The PubMed strategy was translated for other databases. All literature database searches were conducted from January to March 2023. To identify other relevant studies, in addition to the articles found in the literature search, we searched Google Scholar, *Journal of Dental Education*, *European Journal of Dental Education*, and we reviewed the reference lists of studies that met the inclusion criteria. The results were limited to English language and included all published articles up to February 2023. The results were aggregated and then deduplicated.

A data mapping form was jointly developed by the reviewers to determine which variables should be extracted from the articles. The following data were then extracted: author, year of publication, name of the journal, corresponding author/country, study design, study objectives (citing who were the underrepresented persons considered in the article), strategies (ideas/plans/actions/methods), what has been done, and challenges, and the major conclusions from the articles. The descriptive synthesis of the results focused on the following two questions: (i) What are the general characteristics of the included studies (such as study design, and proposed objectives)? and (ii) Which strategies have been done?

## Results

**Fig. 1:**
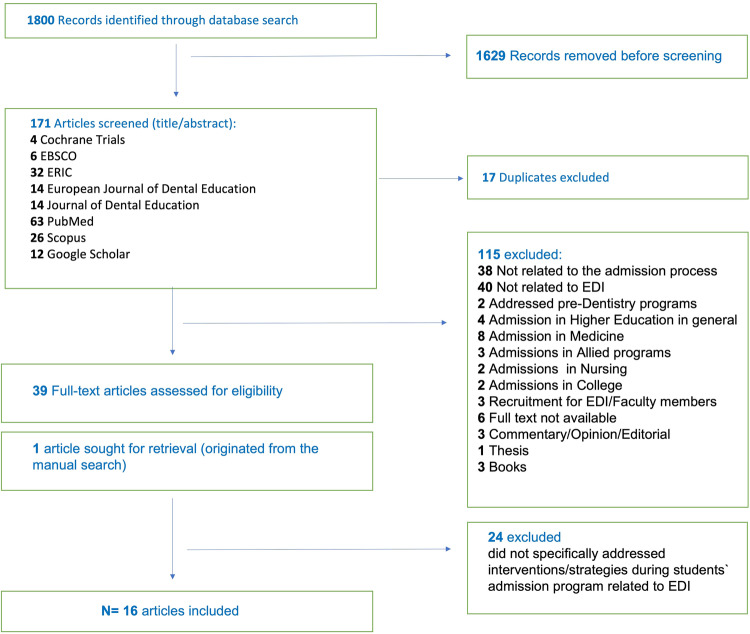
Flow diagram of articles selected for this review. Diagram shows from top to bottom the number of articles screened, excluded (with the reasons for exclusion) and included.

The literature search yielded 1.800 records and 1.629 were removed before screening because they were not related to the topic investigated. From the 171 articles that underwent title/abstract screening, 17 were duplicates and 115 were excluded (reasons for exclusion are shown in the flowchart). Thirty-nine articles underwent full text screening and 24 were excluded because they did not address the strategies used during the admission process. A manual search in the references of relevant studies retrieved one additional article. Therefore, a total of 16 articles were included in this review (Fig. 1).

The 16 articles included [7, 11, 14–27] were published between 2003 and 2022. Publications were higher in number in 2022. The *Journal of Dental Education* was the scientific journal with the highest number of publications (75%). The ranking of the countries with the publications that fitted our inclusion criteria were: United States (n = 12, 75%), Canada (*n* = 1, 6.25%) Scotland, UK (*n* = 1, 6.25%), England, UK (*n* = 1, 6.25%) and Belgium (*n* = 1, 6.25%).

The studies characteristics (author, publication year, journal name, corresponding author/country, study design, and study objectives) are reported in Table 1. Study designs were case studies and critical reviews (*n* = 8, 50%), cross-sectional (including survey and secondary data analysis) (*n* = 5, 31.25%), qualitative methods of analysis (*n* = 2, 12.5%), and retrospective/secondary data collection (*n* = 1, 6.25%).

**Table 1:** General characteristics (author, year of publication, name of the journal, corresponding author/country where the study was conducted, study design, and study objectives) of the articles included in this scoping review (*n* = 16).

Author (year)	Name of the journal	Country of origin	Study design	Study objectives
Ester et al. [23]	Journal of Dental Education	United States	Narrative review and case series	Present strategies for anti-racism and discuss underrepresentation of Black/African American men in dentistry and dental education
Neville et al. [11]	British Dental Journal	England, UK	Cross-sectional (using secondary data analysis)	Illustrate how ethnicity shapes dental education and dentistry in the U.K. (Black-British, Asian-British, and Minority-ethnic)
Fleming et al. [24]	Journal of Dental Education	United States	Qualitative/Focus groups/Thematic analysis	Identify successes, challenges, and opportunities for recruiting Black, Latinx, and American Indian dental students (Black, Latinx, American Indian)
Drazenovich & Mazur et al. [7]	Interchange	Canada	Narrative review and opinion	Provide a human rights-derived methodological framework to help advancing EDI on Canadian university campuses (Indigenous, Islamic people, Immigrants in general, Trans-people)
Cunningham & Kiezebrink et al. [27]	European Journal of Dental Education	Scotland, UK	Qualitative/Individual interviews/Thematic analysis	Explore dental admissions staff beliefs about the quality of different selection tools, and the decision-making in implementing selection practices
Booker et al. [26]	Journal of Dental Education	United States	Narrative review and opinion	Make a critical assessment for implementation of holistic admission practices in dental education
Hewlett et al. [25]	Journal of Dental Education	United States	Narrative review	Identify best practices for increasing enrollment of historically underrepresented minority groups in dental school (racial/ethnic, Black)
Chaviano-Moran et al. [22]	Journal of Dental Education	United States	Cross-sectional (using secondary data analysis)	Assess unintended selection bias with holistic review process and assess the influence of metrics in one dental school
Claeys-Kulik et al. [21]	European University Association report	Belgium	Cross-sectional (survey)	Support European universities in developing strategies towards EDI. Promote dialog between stakeholders at the system level to ensure that regulatory and funding frameworks empower universities to fulfill their social responsibility
Aalboe et al. [20]	Journal of Dental Education	United States	Cross-sectional (using secondary data analysis)	Examine whether underrepresented minority students applying late in the application cycle contributes to their lower enrollment (African American, Hispanic/Latino)
Wilson et al. [19]	Journal of Dental Education	United States	Narrative review and case report	Describes an assessment model that used non-cognitive variables in one dental school admission process
Wells et al. [18]	Journal of Dental Education	United States	Cross-sectional (report)	Explore the rationale for the development of ADEA Admissions Committee Workshop. Track underrepresented minority students‘ enrollment in dental schools where the workshop was presented
Price & Grant-Mills et al. [17]	Journal of Dental Education	United States	Narrative review and case series	Describe the institutional and policy-level strategies that dental schools participating in the Pipeline program used to modify their admissions practices to increase the diversity of their student bodies (African Americans, Hispanics/Latinos, and American Indians/Native Americans)
Lopez et al. [16]	Journal of Dental Education	United States	Retrospective	Identify applicants who should be invited for an interview and to assess applications including non-academic factors in a consistent and systematic manner (underserved, rural, African American, Hispanic, and American Indian)
Price et al. [15]	Journal of Dental Education	United States	Narrative review and longitudinal survey	Demonstrate that a workshop on diversity in admissions can modify the perceptions of individuals involved in the student recruitment and admissions processes and lead to increased matriculation of underrepresented minority students (African American, Hispanic, and American Indian)
Formicola et al. [14]	Journal of Dental Education	United States	Narrative review	Describe how one dental school approached diversity to increase the enrollment of underrepresented minority students (African/American and Latinos)

Not all included articles had as their primary objectives the description and analysis of the strategies used to increase diversity during the admission process. However, they focused on anti-racism actions, and other ideas and approaches, that included best practices addressed to meeting the EDI requirements [7, 11, 14, 21, 23–25]. The articles that addressed dental school admissions as their main concept were straightforward to find information, and also presented insightful statements regarding EDI during admission/selection [15–20, 22, 26, 27]. Overall, the included articles emphasized the holistic review process as one of the most convenient strategy used to increase the enrollment of underrepresented minority groups. Overall, the included articles described diversity and underrepresented minority groups as: Asian-British, Black-British, Black, African American, Black/African American men, Women, Black-women, Hispanic/Latino, Latinx, American Indian, Native American, Indigenous, Islamic persons, Immigrants and Trans people (find information in Table 1).

We also found some interesting insights from the excluded articles. For example, one article [28] described alternative methods of admission in dental education that could support a broadened participation in dental education; however, their study did not specifically focus on EDI issues. Conversely, some studies addressed the need to increase diversity and presented some ideas for recruitment and retention, such as loans, scholarships, and supporting test preparation [5, 20–37]; however, the admission process was not the focus of those studies as well. Ultimately, we agreed to exclude studies that had the objective of reporting workshops and training sessions for admissions committee members [8, 38–40] and had occurred outside the admissions period.

Table 2 describes the strategies used by dental schools to recruit and increase the number of underrepresented minorities during their BSC, DDS and DMD (undergraduate) admission process. The sub-sections below summarize the results.

**Table 2:** strategies related to Equity, Diversity, and Inclusion (EDI) used by dentistry schools during the students‘ admission process – reported in the included articles (*N* = 16).

Strategy	Number of articles that mentioned the strategy/intervention	Authors (Year)
Considering non-cognitive indicators	*n* = 12	Formicola et al. [14], Price et al. [15], Lopez et al. [16], Price & Grant-Mills et al. [17], Wells et al. [18], Wilson et al. [19], Hewlett et al. [25], Booker et al. [26], Cunningham & Kiezebrink et al. [27], Drazenovich & Mazur et al. [7], Fleming et al. [24]
Balancing non-cognitive indicators with metrics may prevent unintended selection bias	*n* = 1	Chaviano-Moran et al. [22]
Focusing on one or two dimensions of diversity at a time	*n* = 1	Claeys-Kulik et al. [21]
Avoiding overreliance in the traditional/quantitative tests (GPA, DAT, etc.)	*n* = 2	Price & Grant-Mills et al. [17], Booker et al. [26]
Collecting individualized information from applicants such as socioeconomic status, and ethnicity	*n* = 3	Booker et al. [26], Drazenovich & Mazur et al. [7], Neville et al. [11]
Creating of an instrument to assist admission committee members to standardize non-cognitive/non-academic indicators for a holistic review	*n* = 2	Lopez et al. [16], Booker et al. [26]
Improving interviewing skills of committee members	*n* = 2	Formicola et al. [14], Wells et al. [18]
Increasing time for individual interviews	*n* = 1	Price et al. [15]
Considering the intersectionality of diverse people	*n* = 2	Claeys-Kulik et al. [21], Neville et al. [11]
Decreasing number of domestic students	*n* = 1	Claeys-Kulik et al. [21]
Opening for international students	*n* = 1	Claeys-Kulik et al. [21]
Using non-structured interviews	*n* = 1	Wilson et al. [19]
Two independent interviewers (at least) for each applicant	*n* = 1	Price et al. [15]
Creation of a Minority Admissions Subcommittee	*n* = 1	Formicola et al. [14]
Professionalising the Admissions Lead Role	*n* = 1	Cunningham & Kiezebrink et al. [27]
Partnership between Admissions Committee and other working groups	*n* = 1	Claeys-Kulik et al. [21]
Support from leadership at school level (in particular, the Dean)	*n* = 7	Formicola et al. [14], Price et al. [15], Price & Grant-Mills et al. [17], Wells et al. [18], Claeys-Kulik et al. [21], Fleming et al. [24], Neville et al. [11]
Changes in the leadership (Dean and Academic Deans): prioritize race and gender	*n* = 2	Wells et al. [18], Ester et al. [23]
Support from leadership at university level	*n* = 3	Formicola et al. [14], Claeys-Kulik et al. [21], Fleming et al. [24]
Relying on support from minority faculty members	*n* = 3	Formicola et al. [14], Claeys-Kulik et al. [21], Ester et al. [23]
Climate study	*n* = 1	Formicola et al. [14]
Re-thinking policies and procedures	*n* = 2	Formicola et al. [14], Drazenovich & Mazur et al. [7]
Incorporating clear statements in the academic policies	*n* = 4	Price & Grant-Mills et al. [17], Claeys-Kulik et al. [21], Hewlett et al. [25], Drazenovich & Mazur et al. [7]
Incorporating students and staff opinions in the university’s strategies	*n* = 1	Claeys-Kulik et al. [21]
Development of programs to increase diversity (e.g., U.S.A. and U.K. Pipeline)	*n* = 6	Price et al. [15], Price & Grant-Mills et al. [17], Wells et al. [18], Wilson et al. [19], Hewlett et al. [25], Neville et al. [11]
Workshops trainings for members of admission and recruitment committees	*n* = 7	Price et al. [15], Price & Grant-Mills et al. [17], Wells et al. [18], Wilson et al. [19], Booker et al. [26], Cunningham & Kiezebrink et al. [27], Ester et al. [23]
Develop an Admission Committee’s mission statement	*n* = 3	Price et al. [15], Price & Grant-Mills et al. [17], Wells et al. [18]
Diversify the Admissions Committee composition	*n* = 4	Price et al. [15], Price & Grant-Mills et al. [17], Wells et al. [18], Ester et al. [23]
Admissions committee should monitor outcomes	*n* = 1	Price & Grant-Mills et al. [17]
Maintain a membership waitlist to fill unexpected vacancies on the admissions committee	*n* = 1	Price & Grant-Mills et al. [17]
Adopt a school’s mission statement focused on diversity and aligned with the university’s	*n* = 1	Price & Grant-Mills et al. [17]
Student financial aid	*n* = 3	Price & Grant-Mills et al. [17], Wells et al. [18], Claeys-Kulik et al. [21]
Student financial aid integrated into the admission process	*n* = 1	Price & Grant-Mills et al. [17]
Institution/School financial investments	*n* = 7	Price & Grant-Mills et al. [17], Wells et al. [18], Fleming et al. [24], Booker et al. [26], Cunningham & Kiezebrink et al. [27], Drazenovich & Mazur et al. [7], Ester et al. [23]
Admissions committee should plan recruitment activities in collaboration with other areas	*n* = 4	Wells et al. [18], Claeys-Kulik et al. [21], Hewlett et al. [25], Drazenovich & Mazur et al. [7]
Provide data on the time of applications submitted by underrepresented minority students	*n* = 1	Aalboe et al. [20]

### Considering the intersectionality of diversity and defining diversity according to the school and society needs

This strategy was considered important because peoples‘ trajectory is characterized by vertical and horizontal segregation. For example, considering gender in isolation may be seen as simplistic and naïve. Other social identities also should be considered. For example, the rising of feminization in dental schools’ admissions needs to be prefaced by the question “which women are being included”? The concept of feminization could lead to the assumption that all women progress easily into dentistry [41]. When, in fact, the number of Black women had not increased compared to the number of White women [7, 11, 21, 24, 41]. Another example is the low number of Black men in dentistry – as they do not have gender privilege, they do not receive the deserved consideration [23].

Institutional commitments to equity aim to create a workforce that meets the population needs [24]. Each school must prioritize their own dimensions of diversity because each school is unique in culture [14]. Literature was strong and consistent in showing that, in some regions of the United States, Black, Latinx, and American Indian need to be favored with opportunities, and need to have some barriers removed to develop a sustainable pathway to enter the dental profession [24, 25]. In Canada, since 2015 there has been an upward surge in international migration – but this has often been met with resistance - mainly when those immigrants come from non-Western countries. Canada has experienced this resistance in the form of Islamophobia and racism [7].

### Considering noncognitive indicators (qualitative factors) in addition to those traditional/quantitative measures (GPA, DAT, etc.) will support a holistic review process

This strategy was reported and strongly recommended by most of the authors. A holistic review process happens when the admissions committee considers an individualized way of assessing the applicants‘ qualifications [19, 26]. Only the traditional/quantitative cognitive measures (GPA, DAT, etc.) may not be accurate predictors for the success of individuals from non-traditional, disadvantaged, and minority groups. The articles recommended to consider noncognitive indicators. It was mentioned that contemplating noncognitive indicators during the admissions decisions may predict the success of all students, and may help those students who experience some form of discrimination [7, 14–19, 24–27]. The following noncognitive variables have been considered in the applicants portfolio: positive self-concept, realistic self-appraisal, overcoming challenges, preferring long range to short-term or immediate needs, leadership experience, volunteerism, employment, participation in community service, and having acquired/developed knowledge in or about a field [42].

### Diversifying the admissions committee composition and providing training to improve their interviewing and assessment skills. Professionalizing the admissions lead role and creating a minority admissions subcommittee. Leaders in the admissions committee would broaden the horizons upon external partnerships. These approaches would require different levels of support from the university and from the dental school

Structural diversity may also involve diversity of age, culture, ethnicity, gender, geographic origin, economic status, and educational experiences [17]. Training was an important intervention to admission committee members and the following were reported: workshops, annual orientation seminars for new and returning members, and feedback mechanisms [15, 17–19, 23, 27].

The idea of professionalizing positions that are focused on diversity (i.e., creating an official leadership position, such as vice-dean, or academic dean) was discussed and implemented in some schools and it was considered a key-factor to increase admissions of racial and ethnic diverse students [14]. Dental schools that experienced the greatest growths in diversity had made rigorous efforts to have an admissions committee and other leadership positions that reflected their members diversity [18]. This had been achieved by including underrepresented dentists/providers, alumni, faculty from other university units, staff, and students in the admissions committees and in other school leadership positions. These diverse leaders would bring a positive outcome on diversity, such as, a possible facilitation of collaborative recruitment activities with other health professionals [7, 18, 21, 25] and their personal trajectories may help in the dissemination of successful diverse stories and diverse role models in social media (in a way that could attract and inform potential students about the dentistry career) [18]. However, these changes must have the commitment from the dental school dean and the university leaders [7, 18, 21, 25].

### Financial investments and re-thinking policies and procedures

Funding is necessary to support EDI practices in dental schools. Higher institutions have invested in EDI action plans, including strategies for recruitment and retention of a diverse student body and workforce (through pathway programs) [23]; other initiatives are the support and creation of EDI services; and the promotion of EDI leadership roles [7]. Financial aid for students (full or partial scholarships) was also seen as an important support for the institution to achieve its mission for diversity [24]. Authors suggested that financial aid information should be integrated into the admissions interview process, and subsequently, information sessions should be offered at regular intervals in the dental curriculum [17].

Previous circumstances (court decisions from lawsuits in North America) have prompted institutional policymakers and admissions committees to critically inspect legal boundaries that shape policies and procedures for improving diversity in dental education. The suggestion coming from the interpretation of those circumstances is that the admissions committee mission statements, related to diversity, need to be aligned with the mission from the institution and school/college [24, 41].

## Discussion

This review provided a collection of EDI initiatives organized across various dental schools in their admissions process. Our analysis agreed that the entire sample of articles offered a claim to advance EDI activities in a manner that is relevant to the dental education community. However, the nature of evidence encountered was mostly descriptive and did not embrace data collection. It is known that background/descriptive information is important and helpful—but it comes influenced by beliefs, opinions, and politics. Most articles (75%) were published in the Journal of Dental Education, and one of the reasons for that was the special issue on EDI and Belonging published in September 2022.

The most reported strategy was the use (or the agreement to use) of noncognitive indicators during the selection of applicants to support a holistic review process (see Table 2) [7, 14–19, 24–27]. Certainly, this strategy is useful to raise awareness in relation to the limitations and barriers that the applicant needed to overcome in life to be able to apply to a dental school. Employment and community service, for instance, may demonstrate dedication and perseverance [26]. However, some noncognitive indicators are considered nonevidence based approaches—and, therefore, have received negative criticism. This is the case of personal statements and reference letters; tools that exhibit low predictive validity [27, 43–45]. The included articles explained that noncognitive indicators can minimize the dependence only on quantitative metrics, that may reflect a fixed mind-set [26]. The dilemma is that, even having a diverse composition in the admissions committee, people need to develop heuristics (or common-sense approaches which individuals rely on to make quick decisions on everyday life). Heuristic approaches depend on factors such as information available, time and cognitive capability of the individual [27, 46]. One of the included articles showed that judgements about effective selection are shaped by previous practices rather than using a more rational decision-making processes – and this was considered a factor that could jeopardize admissions valuable decisions [27].

Ideas to overcome these and other challenges were: (1) to provide workshops and training that help admissions leaders and committee members to develop optimal heuristics, as well as to minimize stereotyping or using mental shortcuts to classify and categorize people [15, 17–19, 23, 26, 27], and (2) to produce research on noncognitive factors with internal validity (low risk of bias) and external validity (applicability) – this production would be independent of the study design [25, 27, 47].

All the above changes need investment, not only monetary but also non-monetary, including ongoing commitment and engagement from the school’s and university’s leaders to help producing a cultural change in favor of EDI [24]. EDI-focused leadership roles would be appointed from central university level, and this would favor the EDI-leader, because the policies would not change or stop when there was a change in the deanship. Rethinking and modifying academic policies and procedures were also stated by the articles.

Strengths of this current review were: the comprehensive literature search that was performed aggregating several databases; the involvement of two investigators in the screening and data extraction process; the ability to identify the type (study designs) of available evidence; the ability to demonstrate that the literature is still incipient in the field of EDI-based approaches in dental education admissions process; the opportunity to clarify key-concepts to understand the subject (intersectionality, noncognitive indicators, holistic approaches etc.); and the ability to identify EDI-related strategies in dental schools admissions. Limitations were the restrictions imposed in our sample, including language, geography, and time - in which may have resulted in bias. All articles were published in English with potential for the risk of publication bias. Other limitations included a possible failure of our search strategy to identify relevant studies for inclusion and the possibility of data extraction errors.

## Conclusions

This review obtained meaningful findings associated with the aim of increasing the proportions of underrepresented minorities/diversity in undergraduate dental academia, such as: paying attention to the intersectionality of diversity to be able to construct a successful holistic review process; and investing in diversification of the academic leadership, providing training for skills improvements during students‘ selection and admissions. The recent increase in published research on EDI-related to the dental admissions process might imply a positive trend in the studied field.

## Data Availability

The authors confirm that the data supporting the findings of this study are available within the article.
